# A simple and robust clinical manufacturing process for the ex-vivo expansion of autologous T cells genetically engineered to express an anti-BCMA chimeric antigen receptor (CAR) for the treatment of multiple myeloma

**DOI:** 10.1186/2051-1426-3-S2-P117

**Published:** 2015-11-04

**Authors:** Alden Ladd, Graham Lilley, Mingli Li, Dan Cossette, Gregory W Hopkins, Dawn Maier, Michael Paglia, Richard A Morgan

**Affiliations:** 1 bluebird bio, Cambridge, MA, USA

## 

Adoptive transfer of autologous T cells genetically engineered to express chimeric antigen receptors (CARs) has emerged as a promising approach for the treatment of cancer. To this end, we have been developing an anti-BMCA CAR T cell approach for the potential treatment of multiple myeloma. B cell maturation antigen (BCMA) is an attractive CAR T cell target since nearly all multiple myeloma tumor cells express BCMA, while normal tissue expression is restricted to plasma cells and a subset of mature B cells. However, to fully realize the therapeutic potential of these engineered T cells will require the development of simple and robust manufacturing processes capable of supporting larger clinical studies as well as the scale up to commercial cell product generation.

bluebird bio's approach has focused on developing a simple and robust closed system manufacturing process that could be readily transferrable to cGMP manufacturing. Briefly, cultures are initiated with autologous PBMC in the presence of anti-CD3 and anti-CD28 antibodies obviating the need for T cell selection. Activated T cells are then efficiently transduced with an anti-BCMA Lentiviral vector by direct inoculation of the culture with the virus. The T cells are then expanded for a relatively short period of 7-10 days in gas permeable culture bags. Finally, cells are harvested, washed and cryopreserved in the final formulation for infusion.

Using the above clinical manufacturing platform, we consistently achieve greater than 6 population doublings in 7-10 days, starting with as little as 100e6 PBMC. Despite using PBMC as input, the purity of the cell product after expansion is >99 % CD3^+^. Moreover, by using our experience in generating clinic ready high titer Lentiviral vectors we are able to consistently achieve >50 % of the T cells expressing the anti-BCMA CAR from a single round of transduction with an average vector copy number of 1.73 ± 0.43 Importantly, the anti-BCMA CAR T cells manufactured using the above platform retain potent anti-tumor activity both in vitro and in vivo.

In summary, the Lentiviral-based CAR T cell manufacturing platform we have established is robust, and results in a high quality cell product. Importantly, this is achieved with fewer complex manipulations representing an important step toward the development of CAR T cell products.

